# Six-year trend and risk factors of unsuccessful pulmonary tuberculosis treatment outcomes in Thai Community Hospital

**DOI:** 10.1186/s13104-021-05504-z

**Published:** 2021-03-09

**Authors:** Sakarn Charoensakulchai, Chaiyapun Lertpheantum, Chanapon Aksornpusitpong, Peeranut Trakulsuk, Boonsub Sakboonyarat, Ram Rangsin, Mathirut Mungthin, Phunlerd Piyaraj

**Affiliations:** 1 Department of Parasitology, Phramongkutklao College of Medicine, 315, Rajawithee Rd, Thung Phyathai Sub-District, Rajadhewi District, Bangkok, 10400 Thailand; 2 Phramongkutklao College of Medicine, 315, Rajawithee Rd, Thung Phyathai Sub-District, Rajadhewi District, Bangkok, 10400 Thailand; 3 Department of Military and Community Medicine, Phramongkutklao College of Medicine, 315 Ratchawithee Rd, Chang Wat, Bangkok, 10400 Thailand

**Keywords:** Risk factors, Pulmonary tuberculosis, Rural health, Treatment

## Abstract

**Objective:**

Tuberculosis (TB) is a major cause of morbidity and mortality globally. Despite efforts to eliminate TB in Thailand, the incidence rate has declined slowly. This study aimed to identify the incidence and risk factors of unsuccessful pulmonary TB treatment (failed, died and loss-to- follow up) in a community hospital in Chachoengsao Province, Thailand from 1st January 2013 to 31st December 2019.

**Results:**

A total of 487 patients were eligible for the study. The incidence of unsuccessful treatment was 21.67/100 population person year. Risk factors of unsuccessful pulmonary TB treatment were unemployment (adjusted hazard ratio (AHR) 3.12, 95%CI 1.41–6.86), HIV co-infection (AHR 2.85, 95%CI 1.25–6.46), previous history of TB (AHR 2.00, 95%CI 1.04–3.81), positive sputum AFB at the end of the intensive phase (AHR 5.66, 95%CI 2.33–13.74), and sputum AFB was not performed at the end of the intensive phase (AHR 18.40, 95%CI 9.85–34.35). This study can be utilized to improve prevention and intervention of TB treatment by strengthening public health system on treatment quality especially TB patient monitoring tools or methods easy for accessing to patients in communities.

**Supplementary Information:**

The online version contains supplementary material available at 10.1186/s13104-021-05504-z.

## Introduction

Tuberculosis (TB) is a major public health problem worldwide [1, 2]. Most cases were in South-East Asia, Africa, and West Pacific [1]. Despite the decline of TB incidence in recent years, the progression was unable to achieve the goal of World Health Organization (WHO) End TB Strategy by 2020 [2].

In Thailand, community hospitals are frontiers in combating TB in rural areas. Community hospitals are public hospitals that emphasize primary and secondary health care [3]. Thus, most of patients’ health care in rural areas of Thailand relies on these hospitals. Challenges in TB treatment in these settings included patient’s poor TB perception and knowledge [4, 5], low socioeconomic status [4], and case management by non-physician health-care providers [6].

Studies of risk factors of unsuccessful pulmonary TB treatment were scarce in community hospitals. This study aimed to identify incidence and risk factors of unsuccessful pulmonary TB treatment outcomes in Thai community hospital setting.

## Main texts

### Method

#### Study population and setting

This study included pulmonary TB patients receiving treatment at outpatient department (OPD) of a community hospital in Chachoengsao Province, Thailand from 1st January 2013 to 31st December 2019. Patients were followed from the date they initiated on TB treatment. This study included only adult patients (age ≥ 18 years at the start of TB treatment) andexcluded patients with extrapulmonary TB without pulmonary TB involvement, latent TB, died from causes other than TB and TB-related illness during TB treatment and transferred to other treatment units. The exclusion of extrapulmonary TB without co-existing pulmonary TB was due to limited cases. The studied hospital was F1 grade, defined by Health Administration Division as a large-sized community hospital with 120 beds with both general practitioner physicians or family physicians and specialists from at least one major specialty.

#### Study design

Retrospective cohort study was conducted from 1st January 2013 to 31st December 2019 to identify incidence and risk factors for unsuccessful pulmonary TB treatment.

#### Data collection

Data stored in tuberculosis treatment card and online registration platform from 2013 to 2019 were retrieved. To counter file lost and incompleteness and verifying number of files against cases notified, data were retrieved from both treatment cards and online platform which were identical. The data were registered into electronic platform and confirmed by double-checking on both sources, patients’ OPD cards and records of chest radiograph. They included baseline characteristics (gender, age, occupation, history of imprisonment and history of TB contact), medical history (co-morbid illnesses, human immunodeficiency virus (HIV) infection, history of previous TB, history of Bacillus Calmette–Guérin (BCG) vaccination), courses of TB treatment (date of treatment initiation and completion of current TB episode, sputum acid-fast bacilli (AFB) at the start of treatment and end of intensive phase, chest radiographs when treatment was initiated, case status, and hepatotoxic side effect following the treatment) and treatment outcomes (cured, completed, failed, died, and loss-to-follow up).

#### Operational definitions

Pulmonary TB is a case of TB involving lung parenchyma [7, 8]. Treatment outcomes can be classified as cured, completed, failed, died, loss-to-follow up, and transferred [8]. Patients are cured when their sputum AFB smear or culture is negative at the end of treatment if their sputum AFB is positive at the start of treatment. Patients are registered completed when their treatment ended without sputum AFB smear or culture results in the last month of treatment result, but the latest sputum smear is negative. Treatment failure is considered when patients’ sputum AFB at the 5th month or later is positive. Patients are classified ‘die’ when they die before treatment completed regardless of causes. Loss-to-follow up is defined as interrupted treatment for at least 2 consecutive months. Patients with cure and complete treatments were grouped as successful treatment. Patients who failed, died and loss-to-follow up were classified as unsuccessful treatment. Definitions of cases and hepatotoxicity were described in Additional file 1.

#### Statistical analysis

This study used STATA 15.1 (Stata Corporation, College Station, TX, USA) for analyzing incidence and risk factors of unsuccessful pulmonary TB treatment (failed, died and loss-to-follow up). Incidence was calculated using person-time function. The incidence rate was per 100 person-year (PY). Risk factors were calculated by Cox proportional hazard model. Univariate cox proportional hazard model was used for analyze risk factors of unsuccessful treatment outcomes. Factors which were significant or had p-value < 0.20 in univariate analysis or significant in previous studies were recruited for multivariate cox proportional hazard model. Factors with p-value ≤ 0.05 were statistically significant.

#### Ethical consideration

This study was approved by Institutional Review Board Royal Thai Army Medical Department. The study number was R054h/62_Exp. Written informed consents were obtained from all participants when they started TB treatment at the treatment site.

### Results

From 519 TB patients, 3 were dropped out due to incomplete treatment initiation or completion dates. Nine were excluded due to extrapulmonary TB without pulmonary TB involvement. Eight had latent TB. Four died or loss-to-follow up before initiation of treatment. Six died from causes other than TB and TB-related illness. Two were transferred to other treatment units. Consequently, 487 patients were enabled for this study.

#### Baseline characteristics

From 2013 to 2019, least patients (2.87%) were registered in 2013 and most (19.10%) were registered in 2016. Majority of the population were male (67.69%), age ≤ 45 (35.99%), and were laborers (64.42%). Patients with HIV, diabetes mellitus (DM), chronic obstructive pulmonary disease (COPD) and extrapulmonary TB were 5.12%, 10.86%, 4.92% and 10.02%, respectively. Patients with previous history of TB and history of TB contact were 85.28% and 72.19%, respectively. Most were new cases (87.53%), smeared positive TB (70.76%) and had reticulonodular infiltration (81.39%). The results were stratified in Table 1.

**Table 1:** Baseline characteristics

Characteristics	n (%)
Year treatment initiated	
2013	14 (2.86)
2014	59 (12.07)
2015	76 (15.54)
2016	94 (19.22)
2017	92 (18.81)
2018	90 (18.40)
2019	64 (13.09)
Gender	
Male	331 (67.69)
Female	158 (32.31)
Age	
≤ 45	176 (35.99)
46–60	164 (33.54)
≥ 60	149 (30.47)
Occupation	
Labor	315 (64.42)
Unemployed	60 (12.27)
Others	114 (23.31)
HIV co-infection (n = 488)^a^	
Yes	25 (5.12)
No	463 (94.88)
DM (n = 488)^a^	
Yes	53 (10.86)
No	435 (89.14)
COPD (n = 488)^a^	
Yes	24 (4.92)
No	464 (95.08)
Co-existing extrapulmonary TB	
Yes	49 (10.02)
No	440 (89.98)
Previous history of TB	
Yes	72 (14.72)
No	417 (85.28)
History of TB contact	
Yes	136 (27.81)
No	353 (72.19)
Case status	
New case	428 (87.53)
Relapsed case	43 (8.79)
Retreated case	18 (3.68)
Tobacco use	
Yes	318 (65.03)
No	171 (34.97)
Alcohol use	
Yes	324 (66.26)
No	165 (33.74)
BCG vaccination	
Yes	411 (84.05)
No	78 (15.95)
History of imprisonment	
Yes	80 (16.36)
No	409 (83.64)
Sputum AFB at diagnosis	
Positive	346 (70.76)
Negative	126 (25.77)
Not performed	17 (3.48)
Chest radiographs findings at diagnosis	
Reticulonodular infiltration	
Yes	398 (81.39)
No	91 (18.61)
Pleural effusion	
Yes	37 (7.57)
No	452 (92.43)
Lung cavity	
Yes	49 (10.02)
No	440 (89.98)
Miliary TB	
Yes	3 (0.61)
No	486 (99.39)

*HIV* human immunodeficiency virus, *DM* diabetes mellitus, *COPD* chronic obstructive disease, *TB* tuberculosis, *BCG* Bacillus Calmette–Guérin, *AFB* acid-fast bacilli

^a^Missing value = 1

#### Treatment follow-up results and outcomes

Following the initiation of TB treatment, patients affected by hepatotoxic side-effect of TB treatment were 10.84%. At the end of the intensive phase, 6.34% of the patients had positive sputum AFB. Two (0.41%) patients were transferred out. Total successful treatment cases were 88.09% which can be classified as 55.03% cured and 33.06% completed. The results were shown in Table 2. Unsuccessful treatment outcome has been declining from 21.43% in 2013 to 9.38% in 2019. Additional file 2: Figure S1 displayed trend of unsuccessful treatment outcomes.

**Table 2:** Treatment follow-up results and outcomes

Treatment follow-up results and outcomes	n (%)
AST elevation	
Normal and < 3 times	441 (90.18)
≥ 3 times	48 (9.82)
ALT elevation	
Normal and < 3 times	462 (94.48)
≥ 3 times	27 (5.52)
Total bilirubin elevation	
< 3 mg/dL	463 (95.07)
≥ 3 mg/dL	24 (4.93)
Hepatotoxicity side effect during treatment	
Yes	53 (10.84)
No	436 (89.16)
Sputum AFB follow up at the end of intensive phase	
Positive	31 (6.34)
Negative	420 (85.89)
Not performed	38 (7.77)
Transferred out to other treatment units	2 (0.41)
Outcome (n = 487)^a^	
Cure	268 (55.03)
Complete	161 (33.06)
Die	40 (8.21)
Loss to follow-up	16 (3.29)
Fail	2 (0.41)

*AST* aspartate aminotransferase, *ALT* alanine aminotransferase, *AFB* acid-fast bacilli

^a^ Two were excluded due to transferred to other treatment units

#### Incidence of unsuccessful treatment outcome

Overall 58 cases were identified after the total follows up time of 267.68 PY which resulted in a cumulative incidence of 21.67 per 100 PY. Incidence rates were high among patients age ≥ 61 (30.96 per 100 PY), unemployed (49.97 per 100 PY), having HIV co-infection (76.95 per 100 PY), had previous the history of TB (47.18 per 100 PY), affected by hepatotoxic anti-TB drug regimen (63.23 per 100 PY) and sputum AFB at end of intensive phase (197.20 per 100 PY). Incidence rates of unsuccessful treatment were highest in 2013 (46.31 per 100 PY) and decreased significantly (p = 0.001) to 15.21 per 100 PY in 2019. Incidence rates and trend of unsuccessful treatment were depicted in Table 3 and Additional file 3: Figure S2 respectively.

**Table 3:** Incidence rates and risk factors of unsuccessful pulmonary TB treatment outcomes in community hospital, 2013–2019

Characteristics	Total population	Unsuccessful	PY of observation	Incidence/100 PY	HR (95% CI)	P-Value	Adjusted HR (95% CI)	P-value
All	487	58	267.68	21.67				
Year treatment initiated								
2013	14	3	6.48	46.31				
2014	59	8	30.91	25.88				
2015	76	10	43.11	23.19				
2016	93	14	50.86	27.53				
2017	92	9	54.59	16.49				
2018	90	9	48.87	18.42				
2019	63	5	32.87	15.21				
Gender								
Male	329	40	183.98	21.74	1.00			
Female	158	18	83.70	21.51	0.98 (0.56–1.72)	0.954		
Age								
≤ 45	176	16	95.93	16.68	1.00		1.00	
46–60	164	17	91.00	18.68	1.10 (0.55–2.18)	0.788	1.01 (0.49–2.09)	0.972
≥ 61	147	25	80.75	30.96	1.79 (0.95–3.36)	0.072	0.88 (0.42–1.88)	0.748
Occupation								
Unemployed	60	15	30.01	49.97	1.99 (0.98–4.04)	0.055	3.12 (1.41–6.86)	0.005*
Labor	315	27	174.84	15.44	0.63 (0.34–1.18)	0.148	0.84 (0.41–1.72)	0.636
Others	112	16	62.82	25.47	1.00		1.00	
HIV co-infection (n = 488)^a^								
Yes	25	9	11.70	76.95	4.11 (2.02–8.42)	< 0.001	2.85 (1.25–6.46)	0.012*
No	461	49	255.30	19.19	1.00		1.00	
DM (n = 488)^a^								
Yes	53	5	30.22	16.55	0.74 (0.30–1.85)	0.521		
No	433	53	236.78	22.38	1.00			
COPD (n = 488)^a^								
Yes	23	4	14.27	28.04	1.36 (0.49–3.77)	0.590		
No	463	54	252.73	21.37	1.00			
Co-existing extrapulmonary TB								
Yes	49	7	29.40	23.81	1.06 (0.47–2.36)	0.888		
No	438	51	238.28	21.40	1.00			
Previous history of TB								
Yes	72	19	40.27	47.180	2.79 (1.60–4.84)	< 0.001	2.00 (1.04–3.81)	0.036*
No	415	39	227.41	17.15	1.00		1.00	
History of TB contact								
Yes	136	13	76.51	16.99	0.70 (0.38–1.29)	0.252	0.55 (0.29–1.07)	0.078
No	351	45	191.17	23.54	1.00		1.00	
Case status								
New case	426	46	231.98	19.83	1.00			
Relapsed case	43	9	23.24	38.73	2.16 (1.05–4.42)	0.036		
Retreated case	18	3	12.46	24.08	1.17 (0.36–3.80)	0.363		
Tobacco use								
Yes	316	37	174.78	21.17	0.94 (0.55–1.60)	0.815		
No	171	21	92.90	22.60	1.00			
Alcohol use								
Yes	322	38	177.31	21.43	0.98 (0.57–1.69)	0.950		
No	165	20	90.37	22.13	1.00			
AST elevation								
≥ 3 times	47	17	243.28	16.86	4.17 (2.37–7.35)	< 0.001		
Normal and < 3 times	440	41	24.40	69.67	1.00			
ALT elevation								
≥ 3 times	26	10	11.20	89.33	4.63 (2.34–9.17)	< 0.001		
Normal and < 3 times	471	48	256.48	18.71	1.00			
Total bilirubin elevation								
≥ 3 mg/dL	24	9	15.04	59.82	3.06 (1.48–6.35)	0.003		
< 3 mg/dL	463	49	252.64	19.40	1.00			
Hepatotoxicity side effect during treatment								
Yes	52	17	26.89	63.23	3.74 (2.12–6.58)	< 0.001	1.94 (0.99–3.81)	0.053
No	435	41	240.79	17.03	1.00		1.00	
BCG vaccination								
Yes	409	45	221.19	20.34	1.00			
No	78	13	46.49	27.96	1.39 (0.75–2.58)	0.302		
History of imprisonment								
Yes	80	9	44.21	20.36	0.90 (0.44–1.83)	0.773		
No	407	49	223.47	21.93	1.00			
Sputum AFB at diagnosis								
Positive	346	38	192.41	19.75	0.76 (0.43–1.35)	0.358	1.43 (0.73–2.78)	0.294
Not performed	17	3	10.06	29.83	1.21 (0.35–4.16)	0.763	2.18 (0.59–8.08)	0.243
Negative	124	17	65.22	26.07	1.00		1.00	
Sputum AFB follow up at the end of intensive phase								
Positive	31	7	17.55	39.88	3.97 (1.70–9.27)	0.001	5.66 (2.33–13.74)	< 0.001*
Not performed	38	27	13.69	197.20	19.78 (11.31–34.59)	< 0.001	18.40 (9.85–34.35)	< 0.001*
Negative	418	24	236.44	10.15	1.00		1.00	
Chest radiographs findings at diagnosis								
Reticulonodular infiltration								
Yes	397	49	214.64	22.83	1.39 (0.68–2.84)	0.364		
No	90	9	53.03	16.97	1.00			
Pleural effusion								
Yes	37	5	21.41	23.35	1.11 (0.44–2.78)	0.827		
No	450	53	246.27	21.52	1.00			
Lung cavity								
Yes	49	4	29.76	13.44	0.55 (0.20–1.52)	0.250	0.42 (0.14–1.23)	0.113
No	438	54	237.92	22.70	1.00		1.00	
Miliary shadow								
Yes	2	0	0.98	0.00	–	–		
No	485	58	266.70	21.75	–			

*HIV* human immunodeficiency virus, *DM* diabetes mellitus, *COPD* chronic obstructive disease, *TB* tuberculosis, *AST* aspartate aminotransferase, *ALT* alanine aminotransferase, *BCG* Bacillus Calmette–Guérin, *AFB* acid-fast bacilli

* Significant value at 95% CI

^a^Missing value = 1

#### Risk factors of unsuccessful treatment outcome

Unemployment (adjusted hazard ratio (AHR) = 3.12, 95%CI 1.41–6.86, p = 0.005), HIV co-infection (AHR = 2.85, 95%CI 1.25–6.46, p = 0.012)previous history of TB (AHR = 2.00, 95%CI 1.04–3.81, p = 0.036), sputum AFB positive at end of intensive phase (AHR = 5.66, 95%CI 2.33–13.74, p < 0.001) and did not received sputum AFB examination at end of intensive phase (AHR = 18.40, 95%CI 9.85–34.35, p < 0.001) were risk factors of unsuccessful treatment.

### Discussion

This study addressed incidence and risk factors of unsuccessful pulmonary TB treatment in a community hospital during 2013–2019 which synchronous with WHO End TB 2020 milestone [9].

This study showed decreasing incidence of unsuccessful treatment outcomes. The success rate of this study was similar to the national success rate reported by WHO [1]. By 2019, the successful treatment rate of this study was at 90.63% which was higher than the overall treatment success rate of Thailand [9]. At this percentage, the successful treatment outcome has exceeded the target expected in 2022 by End TB strategy [9]. This could be attributed to the introduction of effective directly observed treatment, short-course (DOTS) and holistic care to patients [10, 11].

It was observed that in 2013, there were few TB cases, but had highest incidence of unsuccessful treatment due to founding phase of TB clinic. The situation improved in the following years from effective patient monitoring. In this setting, involving health-care volunteers, trained individuals to oversee communities’ health welfare, were employed for DOTS at home, as well as health education for communities. Video-call DOTS were employed in patients who were not convenient in visiting hospitals or living far from the communities. Health-care providers (usually TB clinic nurses) would video-call to these patients in specific time of the day to observe them taking drugs.

This study showed that unemployment was the risk of unsuccessful TB treatment as in previous studies [12, 13]. Unemployment had roles in delayed and interrupted treatment [14, 15]. Patients with unemployment were more likely to have low socioeconomic status and thus, had difficulty accessing TB treatment [16]. Unemployment also linked to homelessness which related to unsatisfied TB treatment outcomes [14, 17].

This study demonstrated HIV co-infection as the risk factor of poor TB treatment outcome as explained in various studies [18–20]. This study also showed that incidence of unsuccessful treatment was high among HIV co-infected patients which could be resulted from high mortality rates as 20.00% of TB-HIV patients died, slightly higher than national mortality rate [1, 8, 21]. Thailand aimed on acquired immunodeficiency syndrome (AIDS) elimination by 2030. The plan included easy access to health care services, HIV screening, antiretroviral therapy (ART) and retaining patients in treatment system. The outcome was to make 90% of HIV patients realize they carry the disease, received ART and able to suppress viral load. The outcomes were satisfying in combating HIV, resulting in fewer cases and thus, reducing the chance of opportunistic infections which included TB [22].

Sputum AFB follows up, especially at the end of intensive phase, are important predictor of treatment outcome [23, 24]. This study used follow up of sputum AFB at the end of the intensive phase to identify risk of unsuccessful treatment. Positive sputum AFB during follow up could indicate various factors such as patients’ poor compliance and drug-resistance TB [23, 24]. This study also found that patients who did not received sputum AFB follow up, which contradicted to Thai TB treatment guideline [8], were at risk as well. It could be hypothesized that patients who did not receive proper follow up were either having poor treatment compliance, receiving out-of-track management or missing cases follow-up by health care providers. Out-of-track management and cases missing can be improved by recruiting public health volunteers. Knowledge implementation about case detection and patient monitoring for these volunteers is required to serve health care to communities and reduce workloads on health care providers.

In previous studies, previous treatment of TB was associated with unsuccessful treatment outcomes [6, 25, 26]. Unfavorable previous treatment outcomes are predictive for development of drug-resistance TB in the current treatment [27, 28]. Patients who were loss-to-followed up in previous treatment episodes were more likely to be loss-to-followed up as well in the current treatment (29).

This study highlighted possibility of effective measures to increase yield in the treatment success rate by enforcing networks of the public health care system on TB treatment quality, especially patients monitoring. Active TB case monitoring by local health sectors is the crucial point for encouraging patients to follow and maintain in the treatment system. Developments of methods, tools or technology for active case monitoring and easy to access patients in the communities are requirements which will bring convenience for both patients and health-care workers.

## Limitations

Limitations included registration of tobacco and alcohol consumption in only yes/no form instead of consumption amount and pack-years used Second, the sample size of this study was small due to limited number of patients. Third, this study did not include information on antiretroviral therapy, which would be important predictors of poor TB treatment outcomes.

## Supplementary Information

**Additional file 1.** Operational definitions (TB management, hepatotoxicity, and case definition).

**Additional file 2. Figure S1** Unsuccessful treatment outcomes of pulmonary TB treatment in the community hospital, 2013–2019.

**Additional file 3. Figure S2** Incidence rates of unsuccessful pulmonary TB treatment in the community hospital, 2013–2019.

## Data Availability

The data that support the findings of this study are available from Sanam Chai Kate Hospital and Division of Tuberculosis, Ministry of Public Health but restrictions apply to the availability of these data, which were used under license for the current study, and so are not publicly available. Data are however available from the authors upon reasonable request and with permission of Sanam Chai Kate Hospital and Division of Tuberculosis, Ministry of Public Health.

## Abbreviations

TB: Tuberculosis
AFB: Acid-fast bacilli
HR: Hazard ratio
AHR: Adjusted hazard ratio
OPD: Outpatient department
WHO: World Health Organization
BCG: Bacillus Calmette–Guérin
PY: Person-year
HIV: Human immunodeficiency virus
COPD: Chronic obstructive pulmonary disease
DOTS: Directly observed treatment, short-course
AST: Aspartate aminotransferase
ALT: Alanine aminotransferase
AIDS: Acquired immunodeficiency syndrome
