# Supplementary material for: Six-year trend and risk factors of unsuccessful pulmonary tuberculosis treatment outcomes in Thai Community Hospital
Source: BMC Res Notes. 2021 Mar 9;14:89. doi: 10.1186/s13104-021-05504-z (PMC7941995; doi:10.1186/s13104-021-05504-z)
Supplement: Supplementary file 1 — Additional file 1. Operational definitions (TB management, hepatotoxicity, and case definition). [file 13104_2021_5504_MOESM1_ESM.docx]

**Additional file 1: Operational definitions (TB management, hepatotoxicity and case definition)**

**Description of how TB is managed in this study setting**

According to Thai national tuberculosis control program guideline, screening for TB usually performed in risk groups or patients with suspected symptoms such as chronic cough, fever of unknown origin and unexplained significant weight loss. Laboratory required for diagnosis included chest radiography and sputum AFB as well as additional sputum gene X-pert. After diagnosis of TB, patients will be screened for HIV infection, liver function test, renal function test, eye examination and advice for alcohol drinking cessation. Treatment regimen for adults with new pulmonary TB consist of 2 months of intensive phase (2HRZE) and 4 months of continuation phase (4HR) while patients with other conditions will receive different regimens. Treatment regimen can be altered during the course of treatment according to the monitoring of patients’ conditions. Treatments were usually at set at TB or respiratory infection clinics. Usual follow ups for new pulmonary TB cases were two weeks after treatment initiation, at the end of intensive phase, at the end of the fifth month and at the end of treatment. Patients usually prescribed drugs and had DOTS by either health care volunteers personally at patients’ home, patients’ relatives or video-call from health care volunteers (8). Patients are considered to have smear-positive pulmonary TB when one or more sputum smear specimens at the start of treatment are positive for AFB. Smear-negative pulmonary TB is considered when sputum AFB smear is negative, but culture-positive for M. tuberculosis or a case which clinicians decide to treat with full course of anti-TB therapy with radiographic abnormalities consistent with pulmonary TB with either evidence of HIV infection, or, if HIV-negative, has no improvement in response to a course of broad-spectrum antibiotics excluding anti-TB drugs, fluoroquinolones and aminoglycosides (8).

**Definition of hepatotoxicity**

Hepatotoxicity side effect of anti-TB drugs included symptoms -nausea and vomit several weeks after initiation of drugs - and laboratory results - increase of aspartate aminotransferase (AST)/ alanine amino transferase (ALT) > 3 folds if symptomatic or AST/ALT > 5 folds if asymptomatic (8).

**Case definitions**

Patients are considered to have smear-positive pulmonary TB when one or more sputum smear specimens at the start of treatment are positive for AFB. Smear-negative pulmonary TB is considered when sputum AFB smear is negative, but culture-positive for M. tuberculosis or a case which clinicians decide to treat with full course of anti-TB therapy with radiographic abnormalities consistent with pulmonary TB with either evidence of HIV infection, or, if HIV-negative, has no improvement in response to a course of broad-spectrum antibiotics excluding anti-TB drugs, fluoroquinolones and aminoglycosides.
