# Supplementary material for: Six-year trend and risk factors of unsuccessful pulmonary tuberculosis treatment outcomes in Thai Community Hospital
Source: BMC Res Notes. 2021 Mar 9;14:89. doi: 10.1186/s13104-021-05504-z (PMC7941995; doi:10.1186/s13104-021-05504-z)
Supplement: Supplementary file 2 — Additional file 2. Figure S1 Unsuccessful treatment outcomes of pulmonary TB treatment in the community hospital, 2013–2019. [file 13104_2021_5504_MOESM2_ESM.docx]

**Additional file 2: Figure S1 – Unsuccessful treatment outcomes of pulmonary TB treatment in community hospital,** **2013-2019**

p = 0.001
