# Supplementary material for: Six-year trend and risk factors of unsuccessful pulmonary tuberculosis treatment outcomes in Thai Community Hospital
Source: BMC Res Notes. 2021 Mar 9;14:89. doi: 10.1186/s13104-021-05504-z (PMC7941995; doi:10.1186/s13104-021-05504-z)
Supplement: Supplementary file 3 — Additional file 3. Figure S2 Incidence rates of unsuccessful pulmonary TB treatment in the community hospital, 2013–2019. [file 13104_2021_5504_MOESM3_ESM.docx]

**Additional file 3: Figure S2 – Incidence rates of unsuccessful pulmonary TB treatment in community hospital, 2013-2019**

p = 0.001
